# Telehealth Competencies: Training Physicians for a New Reality?

**DOI:** 10.3390/healthcare12010093

**Published:** 2023-12-31

**Authors:** Ilian Cruz-Panesso, Issam Tanoubi, Pierre Drolet

**Affiliations:** Medical Simulation Centre, Centre d’Apprentissage des Attitudes et Habiletés Cliniques (CAAHC), University of Montreal, Montreal, QC H3T 1J4, Canada; i.tanoubi@umontreal.ca (I.T.); pierre.drolet@umontreal.ca (P.D.)

**Keywords:** telehealth, telehealth competencies, telesimulation

## Abstract

In North America, telehealth increased by 40% between 2019 and 2020 and stabilized at 40% in 2021. As telehealth becomes more common, it is essential to ensure that healthcare providers have the required skills to overcome the challenges and barriers of this new modality of care. While the COVID-19 pandemic has accelerated the design and implementation of telehealth curricula in healthcare education programs, its general adoption is still a major gap and an important barrier to ensuring scaling up and sustainability of the telesshealth practice. Lack of experienced faculty and limited curricular time are two of the most common barriers to expanding telehealth education. Overcoming the barriers of telehealth curricula implementation may require moving away from the classic expert model of learning in which novices learn from experts. As the adoption of telehealth curricula is still in its early stages, institutions may need to plan for faculty development and trainee education at the same time. Questions regarding the timing and content of telehealth education, the interprofessional development of curricula, and the identification of optimal pedagogical methods remain open and crucial. This article reflects on these questions and presents telesimulation as an ideal instructional method for the training of telehealth competencies. Telesimulation can provide opportunities for practical training across a range of telehealth competencies, fostering not only technical proficiency but also communication skills and interprofessional collaboration.

## 1. Introduction

Telehealth, a New Normal without a Roadmap for Teaching and Learning?

Telehealth is a rapidly growing emerging area in medicine [1]. In the United States, telehealth visits increased from 1.4 million visits (1.1%) between 2018 and 2019 to 35 million visits (35.3%) in 2020 [2]. Similarly, in Canada, virtual care increased by 40% between 2019 and 2020 and stabilized at 40% in 2021 [3]. A report from the Canadian Medical Association, in conjunction with other medical regulatory bodies released in February 2022, shows that 94% of physicians use virtual care and that 64% of them will maintain or increase their current level of use [3]. It is, therefore, safe to say that telehealth is here to stay and that its implementation will most likely continue to increase.

Telehealth, also known as virtual care or telemedicine, refers to “any interaction between patients and/or members of their circle of care, occurring remotely, using any forms of communication or information technologies with the aim of facilitating or maximizing the quality and effectiveness of patient care” ([3] p. 3).

The rapid evolution of communication technologies has propelled telehealth, and consequently, a broad array of opportunities of distant care are now possible ranging from asynchronous e-consults, outpatient specialty teleconsultations (synchronous video), telementoring, virtual check-in, remote patient monitoring, and acute virtual care visit. Telehealth is rapidly becoming an essential model of care [4]. Yet, telehealth is considered to be a disruptive and rapid transition without a roadmap [5].

Telehealth has been identified as a priority action plan in the development of global and accessible healthcare systems, even before the pandemic [6,7]; however, its deployment and scaling-up depend on several factors. Some of these factors include technology, sources of funding, and regulatory frameworks that ensure patient safety [3,8,9,10,11]. Research investigating the post-assessment of telemedicine during the pandemic, has shed light on the need to further produce guidelines and protocols for addressing patient management through telemedicine [12]. However, and most importantly, if telehealth is to be the next revolution of the healthcare system, it becomes necessary to consider how to train a skilled workforce able to integrate telehealth into routine care [8]. This article focuses on the training needs of medical students and physicians in practice for whom telemedicine is a new reality. 

Implementation of telehealth curricula might seem an like an evolution (a response to adapting to a change) for those schools that already had a telehealth curriculum and a revolution (a sudden and a violent change) for those who are just beginning to understand how to use it. A study reporting the state of the art of telehealth in education in the U.S. indicates that established medical schools accredited before 2002, are more likely to have integrated telehealth curricula than newer schools [13].

While there is an emergent body of literature aiming to determine telehealth competencies for physicians [3,5,14,15,16,17], the methods of implementing telehealth teaching and learning are still lacking in the academic medicine field [5,18]. The goal of the current perspective is to draw attention to the changes that telehealth brings in practicing medicine, and mainly to reflect on the opportunities and the challenges of integrating telehealth curricula into medical training, which will inevitably be part of the mandate for medical schools to prepare the next generation of physicians for this new reality [18]. We start by presenting the emerging telehealth competency frameworks proposed by the main medical regulatory bodies in the United States and in Canada and discuss how these frameworks can be operationalized in the implementation of a telehealth curriculum from an early integration in the preclinical stages. We also present instructional models for integrating teaching and training of telehealth competencies. We further bring attention to the need to provide telehealth training for both students and medical educators, for whom telehealth is also a new reality. We therefore advocate for a new model away from the classic expert model, in which students and educators have the need to simultaneously learn and develop telehealth competencies. We finally shed light on the use of telesimulation as a future direction for providing telehealth training and assessment. Telesimulation is an ideal instructional tool for reproducing a variety of telehealth situations in a controlled and standardized manner. 

## 2. Methodology

This present perspective paper aligns with the established norms for producing commentary [19]. Commentary and perspective articles are intended to draw attention to current theory and practice [20]. The framework presented in this paper emerged from the best available evidence from the last three years on the topic of telehealth curriculum and telehealth competencies as well as the authors’ (ICP and PD) experience implementing a telesimulation curriculum for large cohorts [21]. While telemedicine emerged mainly during the COVID pandemic, telemedicine curricula are still a gap in the literature. Therefore, the arguments about the applicability of telehealth curricula also emerged from the combined expertise of the authors (more than 10,000 h) in curriculum development and telesimulation (ICP and PD), instructional simulation, and research (IT and PD). 

## 3. Discussion

### 3.1. Telehealth Competencies: An Evolution or a Revolution of the Physicians’ Competencies Landscape?

As telehealth becomes more common, it is essential to ensure that healthcare providers have the required skills to overcome the challenges and barriers of this new modality of care. Telehealth requires different skills than traditional in-person care [16,17,22,23,24]. “Remote encounters require unique communication and technical skills and present interpersonal challenges distinct from traditional in-person visits” [24]. For instance, physicians may need to demonstrate their ability to deescalate difficult patient situations over a remote connection while being professional and using interpersonal and communication skills to provide the best care possible [16]. Digital knowledge and virtual clinical attitudes are some of the emergent competencies that providers need to master to deliver virtual interactions in a safe, relevant, and ethical manner [25]. 

Telehealth brings to the front a paradigm flip in which the doctor comes to the patient and not the other way around. In this sense, telemedicine is an equivalent of a doctor’s home visit, in which the physician has a unique window of opportunity to characterize the patient’s individual context and tailor diagnostic and treatment strategies [26]. This new paradigm puts into evidence that telehealth challenges patient care competencies in all its dimensions (e.g., physical examination, history taking, clinical reasoning, patient management, etc.). Physicians may also be prompted to integrate health data collected via mobile health (mHealth) applications that measure individuals’ health data in everyday life [27]. Data-informed medicine will require physicians to capture, record, and retain health data that facilitates its aggregation, analysis, and inform medical decisions [14]. As a lack of knowledge and digital skills are some of the barriers identified in the physicians’ adoption of and satisfaction towards telehealth [3], it is critical to provide physicians with opportunities for learning and practicing virtual care and data-informed medicine. 

Since the COVID-19 pandemic, different academic and professional regulatory organizations have included emergent telehealth competencies in their standards. The Accreditation Council for Graduate Medical Education (ACGME) [28] updated the physicians’ milestones in 2021 and included digital health as one of the six sub-competencies of patient care milestones. The ACGME describes digital health into an increasing continuum of five acquisition levels, 1 being the lowest level of acquisition and 5 the highest level of acquisition (see Table 1). 

A similar update to medical competencies was conducted by the Association of American Medical Colleges (AAMC) [1], who set telehealth standards to guide schools and teaching hospitals in the design and implementation of curricular and professional development activities. This framework describes six emerging competencies along the continuum of learning starting at the entry to residency to the post-residency stage (see Table 2). 

A forthcoming update to the physician competency framework proposed by the Canadian Royal College of Medicine, CanMEDS, is scheduled for released in 2025. It is anticipated that a substantial modification of all the physician roles will occur to better align with the needs of virtual care [15]. Virtual care (VC) is the preferred term to denominate “provision of individual patient care using technology” [14,15]. This revision involves modifying competencies that already present in the CanMEDS 2015 and introducing new competencies related to the use and integration of technologies in the provision of care. As illustrated in Table 3, the majority of new competencies focus on data-informed medicine skills, encompassing the collection, exchange, aggregation, and analysis data [14]. 

Despite the existence of various emerging telehealth standards, common content areas among them encompass technology skills (e.g., software, troubleshooting difficulties), professionalism (e.g., informed consent and patient privacy), communication skills, physical examination skills in telehealth environments, and the affordance and limitation of telehealth visits [29]. 

Telehealth competencies contribute to establishing common foundational concepts and standards that ensure consistency and a sustainable clinician workforce necessary to deploy high-quality virtual care [13,30]. Rather than constituting a new and independent set of skills, telehealth competencies are proposed as a supplement to existing ones (e.g., entrustable professional activities and milestones) [1,5,16]. 

### 3.2. Telehealth Education: Is There a Teaching and a Training Model Guiding the Design and Implementation Telehealth Curricula?

Like any other set of new skills, telehealth skills and principles need to be taught and trained. Opportunities to acquire and practice telehealth skills are crucial factors associated with physicians’ adoption and satisfaction of telehealth [3]. However, physicians and trainees often receive little or no training to effectively implement telehealth encounters [13,31]. While the COVID-19 pandemic has accelerated the design and implementation of telehealth curricula in healthcare education programs [29], its widespread adoption remains a significant gap and a crucial barrier to ensuring scaling and sustainability of telehealth practice [18,32]. Lack of experienced faculty and limited curricular time are two of the most common barriers to expanding telehealth education [13]. 

There is still a lack of evidence about the teaching and training models guiding telehealth curricula [13,18], and several important questions remain relevant. For instance, at what point in training should telehealth education be integrated? What topics should be addressed and prioritized in telehealth training? What are the best pedagogical methods to teach, train, and assess telehealth competencies [33]? Should telehealth curricula be developed at an interprofessional level? These questions are addressed in the following sections of the present article.

### 3.3. At What Point in Training Should Telehealth Curricula Be Integrated?

The emerging body of literature on teaching and training of telehealth competencies has mainly focused on medical residency and continuing medical education [1,34,35]. However, early integration of telehealth curricula, at the preclinical stage, can positively impact the use and generalization of telehealth practice [13,17,29,35,36]. “Rather than struggling to fit telemedicine into an already overflowing curriculum, medical schools are most successfully able to include telemedicine competencies when they build them into existing components of the curriculum” [35]. For instance, telehealth training can overlap activities already integrated in the curriculum related to rural care, interprofessional training [35], communication, data collection and assessment, professional behavior and patient safety [17]. 

The design of a comprehensive telehealth curriculum requires consideration of various aspects to ensure that healthcare professionals are well-equipped to provide effective VC. Telehealth curricula may be designed to cover a variety of topics; however, it is fundamental that learners acquire fundamental knowledge about the differences between the delivery of traditional and telemedicine care [34]. The telehealth competency frameworks mentioned previously (see Table 1, Table 2 and Table 3) can help to guide the development of learning goals and objectives; however, as with any other curriculum, learning goals must be aligned with a clear understanding of the learners’ needs, the content sequences, resources, and assessment methods [37]. 

A stepwise and longitudinal exposure to telehealth practice can start by conducting patient interviews over live video-streamed [38] and progress to elective telehealth clerkships [39] and interprofessional remote simulations [40]. It is important that telehealth curricula focus not only on practical aspects, such as communication and professionalism, but also that they include opportunities to learn and practice the technical knowledge of telehealth [33], such as how to use telehealth software [41]. Table 4 presents a list of some possible topics to integrate into a telehealth curriculum.

### 3.4. What Are the Instructional Methods for Teaching, Training, and Assessing Telehealth Competencies?

There is still limited evidence about the instructional methods used in the delivery of telehealth curricula and its efficacy [33]; however, teaching and training telehealth competencies can adapt and integrate a variety of instructional strategies, including classical lectures, video/podcasts, peer teaching, and simulation [13] (see Table 5). 

The didactics required to teach telehealth can vary greatly depending on the telehealth modality being targeted and the learning objectives. For instance, if we follow Yaghobian et al.’s [34] stepwise approach for implementing telemedicine training in medical school, we may include telemedicine objectives and terminology in the first year, which can be better aligned with methods such as lectures, videos, and podcasts; then, telemedicine practice behaviors and clinical applications can be introduced in the final 3 years by implementing experiential learning strategies such as simulation, standardized/simulated patient, and roleplay. Finally, telemedicine practice training can be applied in residency rotations, achieved through preceptorship, observation, and gradual participation in virtual patient encounters.

### 3.5. Implications for Educating a New Task Force of Medical Educators

Overcoming the barriers of telehealth curricula implementation may require moving away from the classic expert model of learning, in which novices learn from experts. As the adoption of telehealth curricula is still in its early stages, medical education institutions may need to plan for faculty development and trainee education at the same time [17,45]. There are already some formal programs for training faculty members in different domains, including mental health and cardiology—more specifically, stroke teleassistance. Table 6 describes some examples of continuous professional programs addressing telehealth competencies. 

Telehealth education can also be extended to specific programs addressing rural and underserved communities or even disaster responses [55]. However, this topic is outside the scope of this article. 

## 4. Future Directions 

### Training Physicians Telehealth Competencies Using Telesimulation

Telesimulation, a “direct descendent of telehealth” [10], is an ideal instructional method for teaching and learning telehealth competencies [41,56]. Telesimulation is useful for recreating a variety of telehealth components such as teleconsultation, teleadvice, teleassistance [57], tele-education, telemonitoring [58,59], telerehabilitation [60], telehomecare [61], etc. However, despite the growing literature on telesimulation, there is still a lack of consensus on the definition of the term and its overlapping meaning with other type of distance simulations, such as online, remote, and virtual simulation [10,62]. Common to all these terms is the sematic prefix “tele”, which determines that an action is taken at a distance [63]. Following the definitions provided in the simulation dictionary, edited by the Society for Simulation in Healthcare [64], some differences can be established according the to the time in which simulation occurs (synchronous vs. asynchronous), the simulation tools used, the location of the instructor and the participants, and the type of feedback offered to participants. Table 7 summarizes the definition associated with distance simulation, including telesimulation.

For the purpose of this article, we define telesimulation as follows [64]:

“Telesimulation consists of a communication technology to provide mannequin-based [and/or Distance-based high-fidelity human patient simulation training] simulation education between learners and instructors located remotely from one another. During these sessions, the instructor observes the learners in real time and provides immediate feedback during the debriefing. This platform obviates the need to have instructors, learners, and mannequins in the same place at the same time, potentially allowing simulation-based educational sessions to occur with greater frequency for institutions not located proximate to formal simulation centers” *Healthcare Simulation Dictionary* ([64] p. 52).

Although the previous definition, as stated in the *Healthcare Simulation Dictionary* [64], only considers mannequin-based telesimulation, telesimulation can also be adapted to distance-based simulated patient model to simulate a teleconsultations via videoconferencing tools [21]. In this type of encounter, the healthcare provider connects directly with a simulated patient via video conferencing software to conduct the equivalent of a visit. 

When implementing telesimulation, it is useful to apply some distinctions made in telehealth between telecare and telecure [63,65]. “(1) Telecare—it occurs when someone advances a generic (health-related) request for assistance. A disease is not necessary to evoke such a request, and the other actor is not necessarily a health professional. (2) Telecure—it characterizes the action of taking charge of a specific problem (a disease). Since it implies a specific expertise (curing, treating, or managing a disease), the other actor must be a healthcare professional” ([65] p. 448). 

Telesimulation allows participants to interact at a distance in a safe and standardized environment, beneficiating from timely feedback and individual or interprofessional virtual care scenarios [21]. Telesimulation can provide comprehensive and practical training for several telehealth competencies at the individual and at a team level. For instance, medical students can train communication skills when addressing sensitive issues, such as sexually transmitted infections and confidentiality [21]. Insights on how telesimulation can be effectively integrated into existing medical curricula can be found in another article of the same authors titled “A practical guide for translating in-person simulation curriculum to telesimulation” [21]. In addition, medical students and practitioners can also benefit from exposure to interprofessional work in a virtual environment [66,67], where they are able to consult with other professionals about a case, which—according to the previous definitions [65]—would be considered telecare because the agent of the interaction is not the patient but another healthcare professional. 

An overarching learning objective of simulation-based learning is to optimize the degree of transfer ok knowledge, skills and attitudes to the real clinical environment [68]. One of the contributing aspects of simulation transfer is the level of realism or fidelity of the simulated environment, including the equipment, the setting, and the scenario [69]. However, telesimulation with stimulated patients reproduces the exact conditions of a telehealth consultation and therefore measurements of transfer of skills may not be necessary as the transfer condition is the telesimulation itself [68]. 

Research is still needed to provide insights about the indirect impacts of using telesimulation. For instance, we need research that accounts for learners’ engagement and the positive and negative effects of cognitive load when using multiple communication channels during telesimulation [21].

## 5. Conclusions

The rise of telehealth as a transformative force in healthcare delivery is unmistakable, evident in its exponential growth in the United States and Canada. With telehealth firmly establishing itself as a new norm, it is imperative to address the considerable challenges and gaps in its implementation and integration into medical education. The rapid adoption during the pandemic has emphasized the importance of telehealth competencies for healthcare providers. This paradigm shift requires unique communication and technical skills, distinct from traditional in-person care, emphasizing the need for a skilled workforce capable of navigating this evolving modality.

While telehealth competencies are gaining recognition and have been incorporated into the standards of medical regulatory bodies, the implementation of telehealth curricula remains a complex task. Gaps persist in understanding the optimal timing for integration into medical training, prioritization of topics, and identification of effective pedagogical methods.

Notably, the emergent telehealth competencies contribute to the foundational concepts and standards needed for a consistent and sustainable clinician workforce in virtual care. The competency frameworks proposed by organizations such as ACGME and AAMC lay the groundwork for a systematic approach to telehealth education.

Despite the accelerated efforts in response to the COVID-19 pandemic, challenges in the adoption of telehealth education persist, marked by a scarcity of experienced faculty and limited curricular time. Questions regarding the timing and content of telehealth education, the interprofessional development of curricula, and the identification of optimal pedagogical methods remain open and crucial.

We present telesimulation as an ideal instructional method for telehealth competencies. Defined as a communication technology facilitating simulation education between learners and remote instructors, telesimulation offers a versatile platform for recreating diverse telehealth scenarios. This method, a direct descendant of telehealth, presents an opportunity for practical training across a range of telehealth competencies, fostering not only technical proficiency but also communication skills and interprofessional collaboration.

As we advocate for the incorporation of telesimulation into medical education, we recognize the need for further research to address gaps in understanding learner engagement and the effects of cognitive load during telesimulation. This holistic approach to telehealth integration, spanning early education stages through telesimulation in later training, aims to equip healthcare professionals with the multifaceted skills required in the dynamic landscape of virtual care.

## 6. Limitations

The present article must be interpreted in the context of several limitations. First, the data provided primarily focus on the telehealth education landscape in the United States and Canada. Telehealth implementation, challenges, and regulations can vary significantly in different countries and healthcare systems. Therefore, the generalizability of the arguments presented in a global context may be limited. Secondly, the present perspective addresses the integration of telehealth curricula in the context of medical education without addressing the variability between clinical settings and medical schools, which could add to the debate on how telehealth curricula might vary according to the context and resources available. Although this article acknowledges the barriers and difficulties associated with telehealth and the ethical aspects of it, there is not an in-depth exploration of these aspects, which could also influence the feasibility of implementing telehealth curricula.

## Figures and Tables

**Table 1:** Patient care: digital health milestones as adapted from the ACGME milestones for accredited residency programs [28].

Level 1	Level 2	Level 3	Level 4	Level 5
Uses electronic health record (EHR) for routine patient care activities.	Expands use of the EHR to include and reconcile secondary data sources in patient care activities.	Effectively uses EHR capabilities in managing acute and chronic care of patients.	Uses the EHR to facilitate achievement of quality targets for patient panels.	Leads improvements to the EHR.
Identifies the required components for a telehealth visit.	Performs assigned telehealth visits using approved technology.	Identifies clinical situations that can be managed through a telehealth visit.	Integrates telehealth effectively into clinical practice for the management of acute and chronic illness.	Develops and innovates new ways to use emerging technologies to augment telehealth visits.

**Table 2:** Telehealth competences as adapted from the AAMC telehealth framework.

Telehealth Competency Domain	Brief Description	Example of Skills for a Recent Medical School Graduate	Example of Skills for an Experienced Faculty Physician
Patient safety and appropriate use of telehealth	Clinicians will understand when and why to use telehealth and how to assess patient readiness, patient safety, practice readiness, and end-user readiness.	Explains to patients and caregivers the uses, limitations, and benefits of telehealth—that is the use of electronic communications technology to provide care at a distance.	Role model and taches how to practice telehealth, mitigate risks of providing care at a distance, and assess methods for improvement
Access and equity in telehealth	Clinicians will understand telehealth delivery that addresses and mitigate cultural biases as well as physician bias for or against telehealth and that accounts for physical and mental disabilities and non-health-related individual and community needs and limitations.	Describes one’s own implicit and explicit biases and their implications when considering telehealth.	Role models and taches how to recognize and mitigate biases during telehealth encounters.
Communication via telehealth	Clinicians will effectively communicate with patients, families, caregivers, and healthcare team members using telehealth modalities. They will also integrate both the transmission and receipt of information with the goal of effective knowledge transfer, professionalism, and understanding within a therapeutic relationship	Develops an effective rapport with patients via real or simulated video visits, attending to eye contact, tone, body language, and nonverbal cues.	Role models and teaches effective rapport-building with patients via video visits, attending to eye contact, tone, body language, and nonverbal cues.
Data collection and assessment via telehealth	Clinicians will obtain and manage clinical information via telehealth to ensure appropriate high-quality care.	Obtains history (from patient, family, and/or caregiver) during a real or simulated telehealth encounter.	Role models and teaches the skills required to obtain a history (from patient, family, and/ or caregiver) during a telehealth encounter and incorporates the information into the management plan.
Technology for telehealth	Clinicians will have basic knowledge of technology needed for the delivery of high-quality telehealth services.	Explains equipment required for conducting care via telehealth at both originating and distant sites.	Able to use, and teach others while using, equipment for the intended service at both originating and distant sites.
Ethical practices and legal requirements for telehealth	Clinicians will understand the federal, state, and local facility practice requirements to meet the minimal standards to deliver healthcare via telehealth. Clinicians will maintain patient privacy while minimizing risk to the clinician and patient during telehealth encounters, putting the patient’s interest first, and preserving or enhancing the doctor-patient relationship.	Describes locally relevant legal and privacy regulations for telehealth.	Role models and complies with legal and privacy regulations for telehealth at the local, state, and federal levels.

**Table 3:** Anticipated changes to the CanMEDS physician competency framework regarding virtual care.

CanMEDS Competency Category	Description in the CanMEDS Framework	Anticipated Modification or Inclusion of Competencies for the CanMEDS 2025
Medical Expert role	As Medical Experts, physicians integrate all of the CanMEDS Roles, applying medical knowledge, clinical skills, and professional values in their provision of high-quality and safe patient-centered care. Medical Expert is the central physician Role in the CanMEDS Framework and defines the physician’s clinical scope of practice.	Developing expertise using virtual tools to provide safe, comprehensive patient care.
Communicator role	As Communicators, physicians form relationships with patients and their families that facilitate the gathering and sharing of essential information for effective healthcare.	Recognizes that communication skills are different in a virtual setting and emphasize that physicians need to be excellent communicators regardless of the use of technology.
Collaborator role	as Collaborators, physicians work effectively with other healthcare professionals to provide safe, high-quality, patient-centered care.	modified to include in-person and virtual collaboration in multidisciplinary teams.
Leader role	As Leaders, physicians engage with others to contribute to a vision of a high-quality healthcare system and take responsibility for the delivery of excellent patient care through their activities as clinicians, administrators, scholars, or teachers.	Highlights how the healthcare system is impacted by virtual care, and how new models of care (including virtual care) can improve upon current approaches to healthcare delivery.
Health Advocate role	As Health Advocates, physicians contribute their expertise and influence as they work with communities or patient populations to improve health. They work with those they serve to determine and understand needs, speak on behalf of others when required, and support the mobilization of resources to effect change.	The Health Advocate role focuses on equitable access to virtual care, how digital health can be leveraged to identify community needs, and touches on media advocacy in the digital age.
Scholar role	As Scholars, physicians demonstrate a lifelong commitment to excellence in practice through continuous learning and by teaching others, evaluating evidence, and contributing to scholarship.	The Scholar role focuses on staying up to date with and using technology and recognizes that due to the internet, healthcare literature is evolving past the traditional journal article.
Professional role	As Professionals, physicians are committed to the health and well-being of individual patients and society through ethical practice, high personal standards of behavior, accountability to the profession and society, physician-led regulation, and maintenance of personal health.	The Professional role includes the skills and safeguarding mechanisms that have only now become necessary, given the increase in the use of virtual care.

**Table 4:** Possible topics to integrate into a telehealth curriculum.

Preserving patient privacy [13,17]
Encounter with patient with sensitive issues or limited ability to engage in virtual visits (e.g., patients with hearing-impaired, low health literacy, limited proficiency in the language being treated) [17]
Consideration of patient willingness and readiness for receiving care via telehealth [29]
Incorporation of patient social supports [17]
Limitations of telehealth [13]
Potential changes to the doctor-patient relationship [13]
Remote communication skills [13]
Different telehealth modalities and technologies [13]
Methods of conducting a remote patient history and physical examination [13]
Potential risk to patient [13]
Good website manners [13]/virtual presence [33]
Issues of equitable access [13]
Telemedicine as a tool for reducing health inequities [42]
Federal, state, and local facility and practice requirements [13]
Preparedness of practices [13]
Common problems/troubleshooting [13]
Video-conference-directed resuscitations [43]
Treatment procedure through telemedicine [44]
Features of telemedicine [44]
Medical liability in telemedicine [44]

**Table 5:** Methods used to teach telehealth, adapted from Khullar et al. [13].

Clinical experience—ambulatory
Lecture
Case-based Learning
Clinical experience—inpatient
Discussion, small group
Simulation
Standardized/Simulated Patient
Preceptorship
Discussion, large group
Team-based learning
Virtual patient
Problem-based learning
Role play
Self-directed learning
Workshop
Video/podcast
Peer teaching
Other

Note: The list of methods used to teach telehealth is organized in descendent order from the most popular ones to the least popular in medical schools in U.S. before the pandemic according to Khullar et al. [13].

**Table 6:** Examples of continuous professional programs addressing telehealth competencies.

Institution	Type of Training	Target Public	Topics Covered
Thomas Jefferson University—National Center for Telehealth Education and research (NCTER) [46]	Telehealth facilitator certificate [47]	Healthcare professions	- Applications of telehealth. - Limitations of telehealth. - Differences between telehealth and standard healthcare delivery. - Applications of outpatient an inpatient telehealth - Telehealth platforms. - Troubleshooting of common telehealth technology issues. - Standards and ethical principles of virtual healthcare. - Interprofessional telehealth. - Telehealth etiquette and telehealth coordination.
University of Delaware [48]	Advanced Telehealth Coordinator Certificate—online course	Clinicians, administrators, managers, healthcare IT, and health-related professions.	- Principles of telehealth. - eICU Telehealth resources. - Telehealth funding and Reimbursement - Telehealth policies. - Telemental Health and Ethics. - Quality. - Telehealth in the Home and Remote Patient Monitoring. - Chronic Diseases Telehealth Clinic applications. - Telehealth operational Considerations. - Telehealth Program Planning. - Telehealth management. - Advanced telehealth coordination.
Telehealth certificate institute [49]	Telemental Health Training Certificate (THTC)	Behavioral health professionals	- History, research and settings of telemental health. - Legal practice of telemental health—licensing laws and federal regulations. - Boundaries of competence when using digital or communication technology. - Clinical, legal and ethical considerations when selecting telemental technology. - Benefits and risks of telemental health. - Development of emergency management plans during a telemental health session. - Patients’ criteria for telemental health services. - Effective video or phone session. - Prepare the client for a video or phone session and technological challenges. - Telemental ethical professional practice. - Technology, assessment, and treatment services to fit specific cultural needs.
Telehealth certificate institute [50]	TeleStroke facilitator/ presenter/ Technician Certificate Training Program	Nurses, medical assistants, or medical techs.	- Formulation of accurate algorithms for the diagnosis of a patient with cerebral ischemic stroke. - Patient maneuvering techniques to enhance TeleStroke encounters. - Uses of stroke scoring scale to minimize morbidity and mortality.
American board of telehealth (ABT) powered by the American heart association (AHA) [51]	Courses: - CORE concepts in telehealth certificate program [52] - Telebehavioral health certificate program [53] - TelePrimary care certificate program [54]	Medical professionals	- Introduction to telehealth. - Technology. - Telepresence skills and professionalism. - Legal, regulatory & quality. - Licensing, credentialing, and privileging. - Reimbursement. - Ethical considerations.

**Table 7:** Differences of the distance simulation terms as they are defined in the *Healthcare Simulation Dictionary*, version 2.1.

		Distance Simulation	Online Simulation	Remote-Controlled Simulation	Telesimulation
**Time.** Is the simulation occurring in a synchronous or asynchronous manner?	Real-time (synchronous)	X	X	X	X
	Off-line (asynchronous)		X		
**Simulation tools.** What are the simulation tools or methods used?	Mannequin-based training	X		X	X
	Human patient simulation				X
	Virtual patient in a virtual world		X		
**Simulation operated by**	Participants	X	X	X	X
	Instructors			X	X
	Virtual instructor in a virtual world		X		
**Instructor’s** **(location)** Where is the simulation instructor located?	On-site	X		X	
	Off-site	X		X	X
**Participant’s (location)**	On-site			X	
	Off-site	X	X		X
**Feedback given by**	Instructor	X		X	X
	Virtual instructor on a virtual world		X		
	Human patient				X
	Mannequin			X	X
	Automated platform		X		
	Off-site	X	X		X
